# Clinical characteristics and factors associated with coronary stenosis proximal to a myocardial bridge: a retrospective study

**DOI:** 10.1186/s12872-020-01655-2

**Published:** 2020-08-14

**Authors:** Wen Gao, Jiaxi Zhang, Fei Duan, Shujun Guo, Chun Chen, Liping Du, Jianquan Zhao, Zhihong Zhou

**Affiliations:** 1 First Department of Cardiology, Bayannaoer City Hospital, No. 98 Wulan Buhe Road, Linhe District, Bayannaoer City, 015000 Inner Mongolia Autonomous Region China; 2 Department of Cardiac Rehabilitation, Bayannaoer City Hospital, Bayannaoer City, Inner Mongolia Autonomous Region China; 3 Department of Vascular Abdominal Wall Hernia Surgery, Bayannaoer City Hospital, Bayannaoer City, Inner Mongolia Autonomous Region China

**Keywords:** Myocardial bridging, Stenosis, Left anterior descending artery, Cardiovascular risk

## Abstract

**Background:**

The association of myocardial bridge (MB) with cardiovascular risk and the possible cardiovascular risk factors remain unclear. This study aimed to explore the clinical characteristics and related factors of coronary stenosis proximal to an MB.

**Methods:**

This was a retrospective study of patients with symptoms of coronary atherosclerotic heart disease admitted between 10/2011 and 12/2014 to the Emergency and Cardiology Department of Bayannur Hospital, who underwent selective coronary angiography (SCAG). The patients were assigned to the non-stenosis and stenosis groups according to whether coronary stenosis was proximal to the MB.

**Results:**

Among 244 patients with MB and cardiovascular symptoms, 91 (37.3%) had stenosis proximal to the MB. Compared with the non-stenosis group, there were more males (80.2% vs. 55.6%, *P* < 0.001) and smokers (including those who had quit smoking) (*P* < 0.001) in the stenosis group. There were no significant differences in blood lipid-related indexes (TG, TC, HDL-C, LDL-C, and VLDL-C) between the two groups. Multivariable analysis suggested that MB location in the middle distal or distal segment of the left anterior descending artery (LAD) increased the odds of coronary stenosis proximal to the MB (OR = 0.439, 95% CI: 1.57–7.532, *P* = 0.002), which was then considered an independent factor associated with coronary stenosis proximal to the MB.

**Conclusions:**

In patients diagnosed with an MB by SCAG, only MB located in the middle distal or distal segment of the LAD is independently associated with coronary stenosis proximal to the MB.

## Background

A myocardial bridge (MB) is a common congenital anatomic variation characterized by the left anterior descending artery (LAD) partly covered by the myocardial tissue [1, 2]. An MB can be identified by a combination of imaging modalities such as multidetector computed tomography (CT), CT angiography, intravascular ultrasound, and coronary digital subtraction angiography (DSA) [3–6]. The actual prevalence of MB is unknown because it is often an incidental finding, and its detection varies among imaging methods [7]. Necropsy studies reported widely variable rates of 5–86% [8, 9], but the actual prevalence should be around 25% [7]. Thick MBs, very thick MBs, and very thick MBs with myocardial hypertrophy can be potentially symptomatic, causing myocardial steal, coronary spasm, coronary artery disease, and coronary dissection [7]. Symptomatic patients can be managed conservatively or by percutaneous or surgical treatment [7].

A recent study suggested that the MB is not associated with the risk of cardiovascular (CV) death or myocardial infarction (MI) [10], but this finding remains controversial [11]. Studies suggested that the MB shifts the local hemodynamics toward an increased CV risk [2, 12, 13], while other reports indicated that individuals with or without an MB have similar CV risk [3, 14–18]. Nevertheless, irrespective of the CV risk, it is well recognized that the LAD under an MB or distal to the MB remains free of atherosclerosis, while the proximal part of the MB is vulnerable to the development of atherosclerotic plaques and stenosis [2, 4, 12–14, 19, 20]. A recent study indicated that an MB might increase the risk of atherosclerosis in the LAD proximally to the MB, a risk particularly elevated in patients with risk factors for CV diseases (CVD), including age, male sex, smoking, diabetes, hypertension, hypercholesterolemia, and a family history of CVD [21]. Sun et al. [22] suggested that MB promotes proximal atherosclerosis, without increasing the risk of CVD.

Many previous studies that examined the CV risk of MBs had mixed populations with and without CV risk factors [2, 12–18, 23]. Akishima-Fukasawa et al. [21] stratified patients based on the presence of at least one CV risk factor vs. none, without exploring the associations of specific risk factors. By univariable analysis, Sun et al. [22] showed that atherosclerosis proximal to the MB is more prevalent in males, smokers, and patients with dyslipidemia, hypertension, or diabetes. Nakaura et al. [4] showed that age, diabetes, and an MB in the mid-LAD are independently associated with proximal atherosclerosis.

Hence, the association of the MB with CV risk and the possible CV risk factors remains unclear. Therefore, the aim of the present study was to examine the clinical characteristics and related factors of coronary stenosis proximal to an MB. This study assessed risk factors that could predict a poorer prognosis in patients with an MB, identifying individuals that should be more closely followed and treated.

## Methods

### Study design and patients

This was a retrospective study of patients with symptoms of coronary atherosclerotic heart disease such as chest tightness, palpitation, discomfort of the anterior chest, and retrosternal pain, who were admitted between October 2011 and December 2014 to the Emergency and Cardiology Department of Bayannur Hospital and underwent selective coronary angiography (SCAG). This study was approved by the Ethics Committee of Bayannur Hospital. Informed consent was waived because of the retrospective nature of this study.

Inclusion criteria were: 1) SCAG at Bayannur Hospital; and 2) confirmed MB irrespective of the heart status (acute MI [AMI], angina pectoris, or not). Individuals with incomplete datasets were excluded.

### Diagnostic criteria

The diagnosis of MB was performed by SCAG [24–26]. The whole cardiac cycle was observed for each coronary segment. A coronary segmental stenosis (> 50%) that occurred only during the systole and recovered during the diastole suggested the presence of an MB. The presence of the “half-moon” phenomenon, i.e., half-moon appearance in bridged segments persisting throughout the cardiac cycle but not found in the proximal and distal segments, is highly specific to the MB. In case of SCAG revealing a segment of the coronary artery with stenosis ≥30% during the systole in at least two projection angles, with recovery during the diastole, representing a “milking effect”, an MB with mural coronary artery (MB-MCA) was diagnosed.

Patients presenting with AMI and an MB revealed by SCAG at admission were diagnosed with MB complicated with AMI. The diagnostic criteria for AMI referred to the Guidelines for Management of Acute Myocardial Infarction by the Chinese Society of Cardiology of Chinese Medical Association in 2001 (two of the following three criteria must be met: a) clinical medical history of ischemic chest pain; b) electrocardiogram [ECG] showing dynamic changes; and c) dynamic changes in the concentrations of serum cardiac markers for myocardial necrosis). Patients with typical symptoms of angina pectoris on admission and coronary angiography showing coronary stenosis > 50% with MB were diagnosed with MB complicated with angina pectoris. Patients with coronary angiography after admission showing no significant coronary stenosis, and only MB were diagnosed with simple MB.

### Coronary angiography

After admission, 12-lead ECG, echocardiography, chest X-ray, complete blood count (CBC), urine routine test, blood glucose and lipid level assessment, liver and renal function tests, myocardial enzyme evaluation, coagulation function test, and blood culture were routinely performed to rule out contraindications for coronary angiography, including liver and/or renal dysfunction, bleeding and infection. In addition, the patients were asked for possible allergies to lidocaine and/or contrast media.

An INNOVA3100 digital subtraction angiography system (GE Healthcare, Waukesha, WI, USA) was used. All patients underwent coronary angiography through the radial or femoral artery. After successful puncture, 5000 IU of heparin was injected through the sheath if using the radial way; in case of femoral way use, 2000 IU of heparin was injected. Left and right coronary angiography and multi-axial imaging were performed by the Judkin method.

Angiography data were assessed by two senior physicians. Coronary stenosis was evaluated by the conventional method, based on visual lumen diameter. The stenosis degree was calculated as (length of the normal vascular diameter in the proximal stenosis segment - the stenosis region) /length of normal vascular diameter in the proximal stenosis segment × 100%. Stenosis degree ≤50% indicated no or mild stenosis; stenosis of 51–75% was moderate, and 76–100% was severe stenosis or occlusion. Stent implantation was performed for patients with severe stenosis.

### Grouping

Based on SCAG results, all patients were assigned to the non-stenosis and stenosis groups according to whether there was a coronary stenosis proximal to the MB.

### Observation parameters

Patient baseline data were obtained from medical charts, including age, sex, history of smoking, hypertension, and diabetes. The diagnostic criteria for hypertension were based on the Guideline for the Prevention, Detection, Evaluation, and Management of High Blood Pressure in Adults [27]: systolic pressure ≥ 130 and/or diastolic pressure ≥ 80 mmHg, or current use of antihypertensive drugs. The diagnostic criteria for diabetes were [28]: typical symptoms (polydipsia, polyuria, and unexplained weight loss), and random plasma glucose ≥11.1 mmol/L and/or fasting (at least 8 h) plasma glucose ≥7.0 mmol/L; 2-h plasma glucose in the oral glucose tolerance test, and two consecutive fasting plasma glucose and/or random (postprandial) plasma glucose ≥11.1 mmol/L in patients without typical symptoms; or diabetic patients taking hypoglycemic drugs or insulin injections.

Factors possibly related to proximal coronary stenosis were recorded, including blood lipid indexes such as triglycerides (TG), cholesterol (TC), high-density lipoprotein cholesterol (HDL-C), low-density lipoprotein cholesterol (LDL-C), and very-low-density lipoprotein cholesterol (VLDL-C); coronary angiography data (diagnosis and location of the MB); and stent implantation status after SCAG.

### Statistical analysis

SPSS 22.0 (IBM, Armonk, NY, USA) was used for statistical analysis. Continuous data with normal distribution (based on the Kolmogorov-Smirnov test) were presented as mean ± standard deviation (SD), and those not conforming to normal distribution as median and interquartile ranges. Categorical data were presented as frequency (percentage). Continuous data were analyzed by independent samples t-test (normal distribution) or the Mann-Whitney U test (skewed distribution); categorical data were analyzed by the chi-square test. Multivariable analysis was performed by binary logistic regression with coronary stenosis proximal to the MB or not as the dependent variable. Variables with *P* < 0.05 in univariable analyses were included as independent variables in multivariate analysis. The results were presented as odds ratios (ORs) and 95% confidence intervals (CIs). Two-sided *P*-values < 0.05 were considered statistically significant.

## Results

### Characteristics of the patients

During the study period, 2203 patients underwent SCAG, and 244 with confirmed MB were included in this analysis. Their characteristics are shown in Table 1. Among the 244 patients with an MB and cardiovascular symptoms, 91 (37.3%) had stenosis proximal to the MB. Compared with the non-stenosis group, there were more males (80.2% vs. 55.6%, *P* < 0.001) and smokers (including those who had quit smoking) (*P* < 0.001) in the stenosis group. There were no significant differences in age, hypertension, and diabetes between the two groups.

**Table 1:** Characteristics of the patients

Characteristics	All (*n* = 244)	Non-stenosis group (*n* = 153)	Stenosis group (*n* = 91)	*P*
Age, years, mean ± SD	55.7 ± 9.5	55.2 ± 9.7	56.6 ± 9.1	0.264
Sex, male, n (%)	158 (64.8)	85 (55.6)	73 (80.2)	< 0.001
Smoking, n (%)				< 0.001
No	129 (52.9)	97 (63.4)	32 (35.2)	
Current	90 (36.9)	45 (29.4)	45 (49.5)	
Quit	25 (10.3)	11 (7.2)	14 (15.4)	
Hypertension, n (%)	92 (37.7)	60 (39.2)	32 (35.2)	0.528
Diabetes, n (%)	15 (6.2)	10 (6.5)	5 (5.5)	0.743

Table 2 presents the clinical characteristics of the patients. There were no significant differences in blood lipid-related indexes (TG, TC, HDL-C, LDL-C, and VLDL-C) between the two groups. Patients in the stenosis group were complicated with angina pectoris (63.7%) or AMI (36.3%), and these frequencies were significantly higher than those of the non-stenosis group (43.1% with angina pectoris and 11.1% with AMI) (both *P* < 0.001). An MB in the middle segment of the LAD was predominant in both groups but much more in the non-stenosis group compared with the stenosis group (89.5% vs. 59.3%; *P* < 0.001). The proportion of patients who underwent stent implantation after angiography was higher in the stenosis group (33.0% vs. 9.2%, *P* < 0.001).

**Table 2:** Differences in clinical data between the two groups

Characteristics	All (*n* = 244)	Non-stenosis group (*n* = 153)	Stenosis group (*n* = 91)	*P*
Triglycerides, mmol/L, median (IQR)	1.65 (1.19,2.55)	1.52 (1.16,2.43)	1.705 (1.285,2.805)	0.146
Cholesterol, mmol/L, median (IQR)	4.51 (3.75,5.27)	4.45 (3.71,5.15)	4.55 (3.79,5.44)	0.523
High-density lipoprotein, mmol/L, median (IQR)	0.93 (0.81,1.11)	0.92 (0.81,1.14)	0.94 (0.805,1.105)	0.919
Low-density lipoprotein, mmol/L, median (IQR)	2.58 (2.1,3.27)	2.54 (2.1,3.15)	2.6 (2.115,3.59)	0.198
Very low-density lipoprotein, mmol/L, median (IQR)	0.32 (0.24,0.49)	0.3 (0.23,0.49)	0.34 (0.26,0.52)	0.250
Coronary angiography				
Diagnosis, n (%)				< 0.001
Complicated with angina pectoris	124 (50.8)	66 (43.1)	58 (63.7)	
Complicated with AMI	50 (20.5)	17 (11.1)	33 (36.3)	
Simple myocardial bridge	70 (28.7)	70 (45.8)	0	
Location of the myocardial bridge, n (%)				< 0.001
Proximal + middle proximal segment of LAD	7 (2.9)	2 (1.3)	5 (5.5)	
Middle segment of LAD	191 (78.3)	137 (89.5)	54 (59.3)	
Middle distal + distal segment of the LAD	46 (18.9)	14 (9.2)	32 (35.2)	
Stent implantation after angiography, n (%)	44 (18.0)	14 (9.2)	30 (33.0)	< 0.001

*IQR* Interquartile range, *AMI* Acute myocardial infarction, *LAD* Left anterior descending coronary artery

### Multivariable analysis

**Fig. 1:**
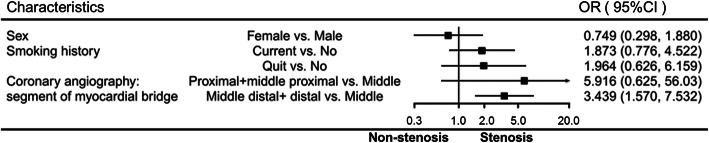
Multivariable analysis of coronary stenosis proximal to myocardial bridge. OR, odds ratios; CI, confidence interval

Multivariate analysis was performed on whether there was a coronary stenosis proximal to the MB, as shown in Table 3 and Fig. 1. Multivariable analysis suggested that MB location in the middle distal or distal segment of the left anterior descending artery (LAD) increased the odds of coronary stenosis proximal to the MB (OR = 0.439, 95% CI: 1.57–7.532, *P* = 0.002), which was then considered an independent factor associated with coronary stenosis proximal to the MB. No other factors were associated with coronary stenosis proximal to the MB.

**Table 3:** Multivariable analysis of coronary stenosis proximal to myocardial bridge

	Univariable analysis			Multivariable analysis		
	OR	95% CI	*P*	OR	95% CI	*P*
Age	1.016	(0.988, 1.044)	0.264			
Sex						
Male	Reference			Reference		
Female	0.308	(0.168, 0.565)	< 0.001	0.749	(0.298, 1.880)	0.538
Smoking history						
No	Reference			Reference		
Current	3.031	(1.706, 5.386)	< 0.001	1.873	(0.776, 4.522)	0.163
Quit	3.858	(1.592, 9.348)	0.003	1.964	(0.626, 6.159)	0.247
Hypertension	0.841	(0.490, 1.441)	0.528			
Diabetes	0.831	(0.275, 2.514)	0.744			
Triglycerides	1.194	(0.960, 1.485)	0.110			
Cholesterol	1.14	(0.885, 1.468)	0.311			
High-density lipoprotein	0.799	(0.290, 2.202)	0.664			
Low-density lipoprotein	1.37	(0.962, 1.951)	0.081			
Very low-density lipoprotein	2.021	(0.678, 6.025)	0.207			
Coronary angiography: location of myocardial bridge						
Proximal + middle proximal segment of LAD	6.343	(1.194, 33.686)	0.030	5.916	(0.625, 56.027)	0.121
Middle segment of LAD	Reference			Reference		
Middle distal + distal segment of the LAD	5.799	(2.872, 11.708)	< 0.001	3.439	(1.570, 7.532)	0.002

*OR* Odds ratios, *CI* Confidence interval, *LAD* Left anterior descending coronary artery

## Discussion

The association of MB with cardiovascular risk and the possible cardiovascular risk factors remain unclear [2, 3, 12–18]. Therefore, this study aimed to examine the clinical characteristics and related factors of coronary stenosis proximal to an MB. The results suggested that in patients diagnosed with an MB by SCAG, only MB location in the middle distal or distal segment of the LAD was independently associated with coronary stenosis proximal to the MB.

In the present study, the frequency of stenosis proximal to the MB among patients with CV symptoms was 37%. In a report by Akishima-Fukasawa et al. [21], among 93 autopsied hearts with MB, 34 (36.6%) showed a stenosis peak around 2.5 cm proximal to the MB, supporting the present findings. In a study by Sun et al. [22], 34.4 and 58.3% of grade I and II-III stenosis cases were proximal to the MB, respectively. In Nakaura et al. [4], 45.7% of the patients who underwent cardiac MDCT for chest symptoms had stenosis proximal to the MB. Ishikawa et al. [23] showed that in patients with MB and AMI, the highest intima-media ratio was observed proximally to the MB. However, CV status was not associated with proximal stenosis in the present study. Therefore, the prevalence of stenosis proximal to the MB in symptomatic patients is high.

As shown above, among classical CV risk factors, only male sex and smoking were associated with stenosis proximal to the MB, while the remaining classical risk factors (age, hypertension, diabetes, and dyslipidemia) showed no associations. Akishima-Fukasawa et al. [21] based their association analysis on the presence of at least one CV risk factor, without distinction among them. On the other hand, Nakaura et al. [4] showed that age and diabetes are independently associated with stenosis proximal to the MB. In a study by Hong et al. [29], age, male sex, diabetes, hypertension, bifurcated vascular lesion, smoking, and hyperlipidemia were found to be associated with proximally located stenosis. Discrepancies among studies might be due to different patient populations and imaging methods.

The present study revealed that the only factor associated with stenosis proximal to the MB was an MB located in the mid-distal or distal LAD, as previously suggested by Nakaura et al. [4]. Since the likelihood of stenosis occurring proximally to the MB is higher than distally [2, 4, 12–14, 19, 20], an MB localized farther along the LAD is too far from the proximal LAD for stenosis development because of hemodynamics change proximally to the MB [2, 12, 13]. Pathological findings indicated that the artery segment proximal to the BM is submitted to hemodynamics that induce pathological changes predisposing the artery to atherosclerosis [2, 30]. Increased shear stress could modulate the expression of genes involved in early atherosclerosis [8, 31, 32].

The present study had some limitations. First, analyses were performed using retrospective data from a single center, with a small sample size. In addition, only patients with CV symptoms were included, and it is unknown whether the obtained results could apply to asymptomatic individuals. Furthermore, this study was not designed to examine the risk of developing CV symptoms or AMI; longitudinal studies are required to address this question. This is of high importance since a number of studies have reported that the presence of an MB is associated with increased CV risk [2, 12, 13] or not [3, 14–18]. Large prospective and multicenter randomized clinical trials are needed to provide high-level evidence.

## Conclusions

In conclusion, among patients with MB and CV symptoms, 37.3% had stenosis proximal to the MB. In individuals diagnosed with an MB by SCAG, only MB location in the middle distal or distal segment of the LAD is independently associated with coronary stenosis proximal to the MB. Therefore, patients with middle distal or distal LAD MB could be at elevated risk of stenosis proximal to the MB and could require closer follow-up and management.

## Data Availability

The datasets used and/or analyzed during the current study are available from the corresponding author on reasonable request.

## Abbreviations

MB: Myocardial bridge
SCAG: Selective coronary angiography
LAD: Left anterior descending artery
CT: Computed tomography
DSA: Digital subtraction angiography
CV: Cardiovascular
CVD: CV diseases
MB-MCA: MB with mural coronary artery
ECG: Electrocardiogram
TG: Triglycerides
TC: Cholesterol
HDL-C: High-density lipoprotein cholesterol
LDL-C: Low-density lipoprotein cholesterol
VLDL-C: Very-low-density lipoprotein cholesterol

## References

[1] Geiringer E (1951). The mural coronary. Am Heart J.

[2] Ishii T, Asuwa N, Masuda S, Ishikawa Y (1998). The effects of a myocardial bridge on coronary atherosclerosis and ischaemia. J Pathol.

[3] Uusitalo V, Saraste A, Pietila M, Kajander S, Bax JJ, Knuuti J (2015). The functional effects of intramural course of coronary arteries and its relation to coronary atherosclerosis. JACC Cardiovasc Imaging.

[4] Nakaura T, Nagayoshi Y, Awai K, Utsunomiya D, Kawano H, Ogawa H (2014). Myocardial bridging is associated with coronary atherosclerosis in the segment proximal to the site of bridging. J Cardiol.

[5] Zeina AR, Odeh M, Blinder J, Rosenschein U, Barmeir E (2007). Myocardial bridge: evaluation on MDCT. AJR Am J Roentgenol.

[6] Yamada R, Tremmel JA, Tanaka S, Lin S, Kobayashi Y, Hollak MB (2016). Functional versus anatomic assessment of myocardial bridging by intravascular ultrasound: impact of arterial compression on proximal atherosclerotic plaque. J Am Heart Assoc.

[7] Tarantini G, Migliore F, Cademartiri F, Fraccaro C, Iliceto S (2016). Left anterior descending artery myocardial bridging: a clinical approach. J Am Coll Cardiol.

[8] Mohlenkamp S, Hort W, Ge J, Erbel R (2002). Update on myocardial bridging. Circulation.

[9] Rossi L, Dander B, Nidasio GP, Arbustini E, Paris B, Vassanelli C (1980). Myocardial bridges and ischemic heart disease. Eur Heart J.

[10] Rubinshtein R, Gaspar T, Lewis BS, Prasad A, Peled N, Halon DA (2013). Long-term prognosis and outcome in patients with a chest pain syndrome and myocardial bridging: a 64-slice coronary computed tomography angiography study. Eur Heart J Cardiovasc Imaging.

[11] Tarantini G, Cademartiri F (2013). Myocardial bridging and prognosis: more evidence but jury still out. Eur Heart J Cardiovasc Imaging.

[12] Ishii T, Hosoda Y, Osaka T, Imai T, Shimada H, Takami A (1986). The significance of myocardial bridge upon atherosclerosis in the left anterior descending coronary artery. J Pathol.

[13] Ishikawa Y, Akasaka Y, Ito K, Akishima Y, Kimura M, Kiguchi H (2006). Significance of anatomical properties of myocardial bridge on atherosclerosis evolution in the left anterior descending coronary artery. Atherosclerosis.

[14] Lee SS, Wu TL (1972). The role of the mural coronary artery in prevention of coronary atherosclerosis. Arch Pathol.

[15] Wang Y, Lv B, Chen J, Zhang Y, Luo F, Lu N (2013). Intramural coronary arterial course is associated with coronary arterial stenosis and prognosis of major cardiac events. Arterioscler Thromb Vasc Biol.

[16] Bayrak F, Degertekin M, Eroglu E, Guneysu T, Sevinc D, Gemici G (2009). Evaluation of myocardial bridges with 64-slice computed tomography coronary angiography. Acta Cardiol.

[17] Juilliere Y, Berder V, Suty-Selton C, Buffet P, Danchin N, Cherrier F (1995). Isolated myocardial bridges with angiographic milking of the left anterior descending coronary artery: a long-term follow-up study. Am Heart J.

[18] Kramer JR, Kitazume H, Proudfit WL, Sones FM (1982). Clinical significance of isolated coronary bridges: benign and frequent condition involving the left anterior descending artery. Am Heart J.

[19] Stolte M, Weis P, Prestele H (1977). Muscle bridges over the left anterior descending coronary artery: their influence on arterial disease (author’s transl). Virchows Arch A Pathol Anat Histol.

[20] Ge J, Erbel R, Gorge G, Haude M, Meyer J (1995). High wall shear stress proximal to myocardial bridging and atherosclerosis: intracoronary ultrasound and pressure measurements. Br Heart J.

[21] Akishima-Fukasawa Y, Ishikawa Y, Mikami T, Akasaka Y, Ishii T (2018). Settlement of Stenotic site and enhancement of risk factor load for atherosclerosis in left anterior descending coronary artery by myocardial bridge. Arterioscler Thromb Vasc Biol.

[22] Sun JL, Huang WM, Guo JH, Li XY, Ma XL, Wang CY (2013). Relationship between myocardial bridging and coronary arteriosclerosis. Cell Biochem Biophys.

[23] Ishikawa Y, Akasaka Y, Suzuki K, Fujiwara M, Ogawa T, Yamazaki K (2009). Anatomic properties of myocardial bridge predisposing to myocardial infarction. Circulation.

[24] Alegria JR, Herrmann J, Holmes DR, Lerman A, Rihal CS (2005). Myocardial bridging. Eur Heart J.

[25] Bourassa MG, Butnaru A, Lesperance J, Tardif JC (2003). Symptomatic myocardial bridges: overview of ischemic mechanisms and current diagnostic and treatment strategies. J Am Coll Cardiol.

[26] Bruschke AV, Veltman CE, de Graaf MA, Vliegen HW (2013). Myocardial bridging: what have we learned in the past and will new diagnostic modalities provide new insights?. Neth Heart J.

[27] Whelton PK, Carey RM, Aronow WS (2018). 2017 ACC/AHA/AAPA/ABC/ACPM/AGS/APhA/ASH/ASPC/NMA/PCNA guideline for the prevention, detection, evaluation, and management of high blood pressure in adults: a report of the American College of Cardiology/American Heart Association Task Force on Clinical Practice Guidelines. Circulation.

[28] Alberti KG, Zimmet PZ. Definition, diagnosis and classification of diabetes mellitus and its complications : report of a WHO consultation. Part 1, Diagnosis and classification of diabetes mellitus. Geneva: World Health Organization; 1999. 10.1002/(SICI)1096-9136(199807)15:7<539::AID-DIA668>3.0.CO;2-S 9686693

[29] Hong H, Wang MS, Liu Q, Shi JC, Ren HM, Xu ZM (2014). Angiographically evident atherosclerotic stenosis associated with myocardial bridging and risk factors for the artery stenosis located proximally to myocardial bridging. Anadolu Kardiyol Derg.

[30] Masuda T, Ishikawa Y, Akasaka Y, Itoh K, Kiguchi H, Ishii T (2001). The effect of myocardial bridging of the coronary artery on vasoactive agents and atherosclerosis localization. J Pathol.

[31] Chatzizisis YS, Coskun AU, Jonas M, Edelman ER, Feldman CL, Stone PH (2007). Role of endothelial shear stress in the natural history of coronary atherosclerosis and vascular remodeling: molecular, cellular, and vascular behavior. J Am Coll Cardiol.

[32] Malek AM, Alper SL, Izumo S (1999). Hemodynamic shear stress and its role in atherosclerosis. JAMA.

